# Factors influencing oncologists’ decisions for germline BRCA testing and adjuvant therapy selection in HER2-negative early breast cancer

**DOI:** 10.3389/fonc.2026.1854965

**Published:** 2026-09-03

**Authors:** Qixin Li, M. Janelle Cambron-Mellott, Xiaoqing Xu, Nicole Kashine-Tack, Jennifer F. Hayes, Kathryn Emily Mishkin, Alexandra Gordon, Oliver Will, Lauren A. Howell, Alejandro Noval, Kathleen Beusterien

**Affiliations:** 1 Oncology Outcomes Research, AstraZeneca, Gaithersburg, MD, United States; 2 Research Services, Oracle Life Sciences, Austin, TX, United States; 3 US Medical Affairs Oncology, AstraZeneca, Gaithersburg, MD, United States; 4 Value & Implementation Outcomes Research, Oncology, Merck & Co., Inc., Rahway, NJ, United States

**Keywords:** adjuvant therapy, BRCA testing, breast cancer, gBRCA, HER2-negative, olaparib, PARP inhibitors, physician preferences

## Abstract

**Introduction:**

With advances in targeted early breast cancer (eBC) treatment, research is needed to understand the factors influencing biomarker testing and respective treatment selection among physicians in routine clinical practice. The purpose of this study was to evaluate factors influencing oncologist decisions for germline BReast Cancer gene (BRCA) mutation (gBRCAm) testing and adjuvant therapy in human epidermal growth factor receptor 2-negative (HER2-) early breast cancer (eBC).

**Materials and methods:**

US oncologists completed an online survey from April to July 2024, which included preference exercises to evaluate the relative importance of select factors for gBRCA testing and adjuvant treatment selection in gBRCAm HER2- eBC.

**Results:**

Among 150 surveyed oncologists, age was over twice as influential as tumor size and lymph node involvement in gBRCA testing decisions for hormone receptor-positive (HR+)/HER2- eBC. Nearly one-quarter reported that gBRCA test results did not impact their treatment decisions. Olaparib-based regimens were the preferred adjuvant treatment for gBRCAm HER2- eBC, over Cyclin-Dependent Kinases 4 and 6 inhibitors alone for HR+/HER2- eBC, immunotherapy or capecitabine alone for triple-negative eBC, and no treatment. Efficacy was the most important treatment attribute. Oncologists reported that patient unwillingness to undergo gBRCA testing or receive adjuvant olaparib due to cost, treatment duration, or other reasons were barriers to treatment.

**Conclusions:**

Despite guideline recommendations, age drives oncologists’ gBRCA testing decisions. To support the preference for olaparib-based adjuvant treatment in gBRCAm HER2- eBC, increased physician awareness of guidelines and the rationale for gBRCAm testing is needed to improve identification of eligible patients.

## 1 Introduction

Early breast cancer (eBC), in which the cancer has not spread beyond the breast or nearby lymph nodes, accounts for over 90% of breast cancer (BC) diagnoses (1), with approximately 80% characterized by low or no overexpression of the human epidermal growth factor receptor 2 protein (HER2-negative [HER2-]) (2). The HER2- subtype is stratified based on hormone receptor (HR) status (3). HR-positive (HR+) tumors express estrogen and/or progesterone receptors, while triple-negative BC (TNBC) lacks estrogen and progesterone receptor expression and exhibits low or no HER2 receptor expression. This stratification is important for guiding treatment decisions for patients with HER2- eBC (3).

In addition to HR status, germline mutations in the *BRCA1* or *BRCA2* genes (gBRCAm) have emerged as key biomarkers that influence both BC risk and treatment decisions (3–5). Women harboring gBRCAm face an increased lifetime risk of developing breast and other cancers. More than 60% of BReast Cancer gene (BRCA) mutation carriers develop breast cancer compared to 13% of non-carriers in general population (4, 5). These individuals are also at elevated risk for developing contralateral BC. Approximately 30% to 40% of *BRCA1* and 25% of *BRCA2* carriers develop a second primary tumor within 20 years (4, 5).

Up to 10% of patients with HER2- BC harbor a gBRCAm (6–11), though prevalence varies by subtype (approximately 15% and 5% in TNBC and HR+/HER2- BC, respectively) (12). While gBRCAm occur less frequently in HR+/HER2-BC, this subtype is approximately six times more prevalent than TNBC and therefore accounts for the majority of gBRCAm carriers (2, 6, 13). These findings highlight the need for tailoring HER2- BC treatment approaches based on a patient’s genetic characteristics.

Historically, adjuvant systemic therapy for HR+/HER2- eBC centered on endocrine therapy, with or without chemotherapy, while treatment for early TNBC (eTNBC) relied solely on chemotherapy (3). However, recurrence remains a significant concern with these traditional approaches; up to 30% of patients with node-positive, high-risk HR+/HER2- eBC recur within five years and approximately 40% of those with eTNBC experience recurrence, often as incurable metastatic disease (14, 15). The limitations of traditional treatment have driven recent advancements in systemic therapy for these patients, which are reshaping the treatment landscape.

Since 2021, several new approvals have transformed the treatment paradigm for patients with high-risk, resectable HER2-eBC in the US. For HR+/HER2- eBC, adjuvant abemaciclib plus endocrine therapy (MonarchE trial) and adjuvant ribociclib plus endocrine therapy (NATALEE trial) are now approved (16, 17). In eTNBC, neoadjuvant pembrolizumab plus chemotherapy followed by post-neoadjuvant pembrolizumab monotherapy (KEYNOTE-522 trial) is approved (18). Additionally, adjuvant olaparib is approved for high-risk patients with gBRCAm HR+/HER2- eBC or eTNBC following neoadjuvant or adjuvant chemotherapy (OlympiA trial) (19), which further segmented the treatment of HER2- eBC based on gBRCAm status. Together, these advancements have introduced a more nuanced approach to HER2- eBC treatment, requiring oncologists to integrate biomarker status and risk stratification into clinical decision-making (20).

In the evolving treatment landscape of eBC, wherein both established and newly approved therapies have their unique attributes, to ensure the optimal treatment reaches the right patients, it becomes critical to understand oncologists’ perspectives on patient management and prescribing behaviors, including disease high-risk categorization and biomarker testing. While international guidelines provide recommendations on who should be referred for gBRCA testing (21), testing rates remain suboptimal (22). Given the recency of these advancements, there is a need to understand the factors influencing oncologists’ decisions in biomarker testing and adjuvant treatment selection in gBRCAm HER2-eBC in routine clinical practice. There may be variability or uncertainty in how oncologists implement guideline-based gBRCA testing and integrate gBRCA results into adjuvant treatment selection and sequencing in routine practice. This study aims to identify the key factors influencing oncologists’ decisions regarding gBRCA testing and adjuvant therapy selection in this patient population.

## 2 Materials and methods

This study involved a cross-sectional internet-based survey of US-based oncologists treating adult patients with HER2- eBC with systemic therapy. Survey content was informed by desk research, qualitative concept elicitation interviews, and input from clinical experts, and refined through cognitive interviews. This study received exemption status from Sterling Institutional Review Board (IRB ID: 11298-EMulvihill) and was conducted in accordance with the Declaration of Helsinki and according to good research practices for conjoint analyses, as recommended by the Professional Society for Health Economics and Outcomes Research (ISPOR) (23). All participants provided electronic informed consent and received fair market value compensation for their participation.

### 2.1 Participants

Our study included US-based oncologists, with a target to survey 50 academic physicians and 100 community physicians. Physicians were recruited via the Survey Healthcare Global (SHG) oncology panel from April to July 2024. SHG maintains a global panel of 2 million physicians and allied healthcare professionals. In the United States, SHG reaches approximately 44% of medical oncologists (mean age: 50 years, 64% male, mean years in practice: 30, mean percent of time in direct patient care: 87%). All panel members must double opt-in before joining SHG’s community. Following enrollment, data verification is conducted using state license records, directories, publicly verified healthcare portals, and the AMA database. Additionally, medical credentials are collected, and enrollees are contacted for verification. Once verified, members are activated and eligible to participate in surveys. Oncology and hematology-oncology specialists registered in the panel were then invited via email to participate in the study.

Eligibility criteria included: age 18 years or older; board-certification or board-eligibility in oncology in the US; practiced in an academic or community setting; spent ≥50% of time in direct patient care; treated ≥25 eBC patients, ≥15 metastatic BC (mBC) patients, and ≥4 TNBC patients in the past 6 months; referred at least 1 BC patient for gBRCA testing in the past 6 months, prescribed poly (ADP-ribose) polymerase (PARP) inhibitors (PARPi), Cyclin-Dependent Kinases 4 and 6 inhibitors (CDK4/6i), or immunotherapy (IO) for HER2- eBC or mBC. Respondents who selected prefer not to answer when asked their age were excluded.

To ensure sample variability, soft quotas were established to recruit oncologists across various demographics, including age, gender, and race/ethnicity, with slight oversampling of Black and Hispanic participants to enhance demographic representation. We also set soft quotas to recruit physicians who have experience prescribing a PARPi, IO, or CDK4/6i in either mBC alone or in both mBC and eBC, with targets of ≥100 physicians for each type of treatment.

### 2.2 Quantitative survey

#### 2.2.1 Survey content

The quantitative survey included direct survey questions (numerical entry, single select, and multi-select questions), three discrete choice experiments (DCEs), and two situational choice experiment (SCE)-based questions. **Supplementary Table 1** contains the survey content.

##### 2.2.1.1 Direct survey questions

Direct survey questions included questions regarding participants’ sociodemographic characteristics (age, gender, race, ethnicity, and state of residence [coded into US Census region]) and practice characteristics (primary practice setting, years of clinical practice experience, time spent in direct patient care, experience prescribing PARPi, IO, and CDK4/6i in BC, locale of primary practice), patient load characteristics (number of eBC, mBC, and TNBC patients seen in past 6 months), and gBRCA testing practices (number of BC patients referred for BRCA testing in past 6 months).

Barriers to gBRCA testing in patients with eBC were assessed with this question: “*Which of the following are reasons for not testing an eBC patient for BRCA?”* Oncologists were able to select one or more reasons, from a predefined list, except for “all eBC patients should be tested for *BRCA*”; if that response was selected, no other reason could be selected.

Oncologists were also asked to report the percentage of patients who received olaparib among their HR+/HER2- eBC patients who are gBRCAm and eligible for olaparib. Oncologists who reported that fewer than 100% of eligible patients received the treatment were then asked: *“For your gBRCA HR+ HER2- eBC patients who are otherwise eligible for Olaparib and did not receive Olaparib, which of the following factors influenced the decision to not prescribe Olaparib?”.* Oncologists could select one or more factors, from a pre-defined list. These questions were repeated with regard to oncologists’ patients with eTNBC.

##### 2.2.1.2 Discrete choice experiments

Three DCEs were used to evaluate the influence of patient characteristics on gBRCA testing decisions and the influence of treatment attributes on adjuvant treatment selection for patients with gBRCAm HR+/HER2- eBC or eTNBC (24–26). A DCE is a widely used preference methodology that captures trade-offs that respondents are willing to make among selected attributes (25). Each DCE presented 5 to 7 attributes with 2 to 3 levels across 9 choice tasks. The attributes and levels included in each DCE are shown in **Table 1**. In the first DCE, oncologists were asked, *“Which HR+ HER2- patient should be more likely to receive a BRCA test at diagnosis? Please assume that these patients have no family history of breast cancer.”* In the second DCE, oncologists were asked, *“Which adjuvant treatment would you be more likely to prescribe to a BRCAm patient with HR+ HER2- eBC? Please assume that IDFS ranges from 86 to 89% and DRFS ranges from 88 to 90% across both treatments.”* In the third DCE, oncologists were asked, “*Which treatment regimen would you be more likely to prescribe to a BRCAm patient with TNBC in early breast cancer?*” Example choice tasks for the three DCEs are shown in **Supplementary Figures 1**, **2A, B**, respectively.

**Table 1:** Question wording, attributes, and levels selected for each DCE and SCE-based question.

DCE on BRCA testing at diagnosis of HR+/HER2- eBC patient with no family history of BC			
Question: Which HR+ HER2- patient should be more likely to receive a BRCA test at diagnosis? Please assume that these patients have no family history of breast cancer.			
Attributes	Level 1	Level 2	Level 3
Age	<35	35-64	≥65
Menopausal status	Pre-menopausal	Post-menopausal	
Lymph node involvement	No lymph node involvement	1 to 3 positive lymph nodes	4 or more positive lymph nodes
Tumor size	Tumor size <1 cm	Tumor size 1–5 cm	Tumor size >5 cm
Breast conservation	Breast conservation is prioritized	Breast conservation is not prioritized	
DCE on adjuvant treatment selection for patient with BRCAm HR+/HER2- eBC			
Question: Which adjuvant treatment would you be more likely to prescribe to a BRCAm patient with HR+ HER2- eBC? Please assume that IDFS ranges from 86-89% and DRFS ranges from 88%-90% across both treatments.			
Attributes	Level 1	Level 2	Level 3
Efficacy	92% OS at 3 years	Unknown OS at 3 years	
Duration	Oral treatment BID for 1 year	Oral treatment BID for 2 years	
Discontinuation rate	10%	19%	
G3/4 Neutropenia	5% risk	19% risk	
G3/4 Diarrhea	<1% risk	8% risk	
G3/4 Anemia	2% risk	9% risk	
All grade Nausea	28% risk	57% risk	
DCE on adjuvant treatment selection for patient with BRCAm with eTNBC			
Question: Which treatment regimen would you be more likely to prescribe to a BRCAm patient with TNBC in early breast cancer?			
Attributes	Level 1	Level 2	Level 3
Efficacy	90% OS at 3 years 87% DRFS at 3 years	79% OS at 5 years 70% IDFS at 5 years	
Treatment administration	Oral treatment	IV treatment	
G3/4 AEs	24% risk	41% risk	77% risk
All grade Diarrhea	18% risk	22% risk	30% risk
G3/4 Immune Related AEs	<1% risk of immune system related side effects that can affect the stomach, intestines, lungs, liver or other organs and require IV steroids and hospitalization	12% risk of immune system related side effects that can affect the stomach, intestines, lungs, liver or other organs and require IV steroids and hospitalization	
G3/4 Hand Foot Syndrome	<1% risk	11% risk	
SCE-based question on patient characteristics influencing adjuvant treatment selection for patient with gBRCAm HR+/HER2- eBC *			
Question: What are you most likely to prescribe as a treatment in the adjuvant setting with or without hormonal therapy for a gBRCAm HR+ HER2- patient with the following characteristics? Please assume the patient received neoadjuvant chemo with non-platinum chemo.			
Treatment options: Olaparib only; CDK4/6i only; Olaparib + CDK4/6i; Olaparib followed by CDK4/6i; CDK4/6i followed by Olaparib; No treatment or hormonal therapy only			
Attributes	Level 1	Level 2	Level 3
Age	45 years	56 years	
Stage	IIB	III	
Ki-67 score	Low	Intermediate	High
pCR status	pCR	no pCR	
Lymph node involvement	No lymph node involvement	1 to 3 positive lymph nodes	4 or more positive lymph nodes
Oncotype Dx score	26	50	
SCE-based Question on Patient Characteristics Influencing Adjuvant Treatment Selection for Patient with gBRCAm eTNBC			
Question: What are you most likely to prescribe as adjuvant therapy for a gBRCAm TNBC patient with the following characteristics? Please assume the patient received neoadjuvant IO plus chemotherapy.			
Treatment options: Olaparib only; IO only; Olaparib + IO; Olaparib followed by IO; IO followed by Olaparib; Capecitabine only; No treatment			
Attributes	Level 1	Level 2	Level 3
Age	45 years	56 years	
Stage	IIB	III	
Ki-67 score	Low	Intermediate	High
pCR status	pCR	no pCR	
Lymph node involvement	≥ pN1	pN0 with ≥ pT2	pN0 < pT2

AE, adverse event; BID, twice a day; BRCA, breast cancer gene; BRCAm, BRCA mutation; DCE, discrete choice experiment; DRFS, distant relapse-free survival; eBC, early breast cancer; eTNBC, early triple-negative breast cancer; G3/4, grade 3/4; HER2-, Human Epidermal Growth Factor Receptor 2-negative; HR+, hormone receptor-positive; IDFS, invasive disease-free survival; IV, intravenous; OS, overall survival.

The experimental design of the DCE was a balanced design with minimal overlap (25). Such a design optimizes overall efficiency in terms of (a) level balance (each level is shown approximately an equal number of times); (b) minimal level overlap (levels repeat within the same task); and (c) orthogonality (levels may be evaluated independently of other levels). All respondents answered a different combination of tasks.

##### 2.2.1.3 Situational choice experiment-based questions

Two sets of questions based on a SCE were used to characterize how patient characteristics influence treatment choice in the adjuvant setting for patients with gBRCAm HR+/HER2- eBC or eTNBC. These questions were included to help characterize the influence, or relative importance, of different patient characteristics on behavior associated with specific treatment options (27, 28).

Each SCE-based question presented a series of 12 patient profiles, with respondents selecting the treatment they would prescribe from 5 or 6 treatment options or choosing no treatment for each profile. The patient profiles varied across 5–6 different patient characteristics. The treatment options, attributes, and levels for each SCE-based question are shown in **Table 1**. In the first SCE-based question, oncologists were asked, “*What are you most likely to prescribe as a treatment in the adjuvant setting with or without hormonal therapy for a gBRCAm HR+/HER2****-*** *patient with the following characteristics? Please assume the patient received neoadjuvant chemo with non-platinum chemo.”* In the second SCE-based question, oncologists were asked, “*What are you most likely to prescribe as adjuvant therapy for a gBRCAm TNBC patient with the following characteristics? Please assume the patient received neoadjuvant immunotherapy (IO) plus chemotherapy.”* Example choice tasks are shown in **Supplementary Figure 3**. The experimental design for which patient characteristics are shown in each profile was orthogonal. Such designs optimize level balance and orthogonality, where no two characteristics (attribute) levels are shown together more often than any other two attribute levels (25).

#### 2.2.2 Survey flow

**Figure 1:**
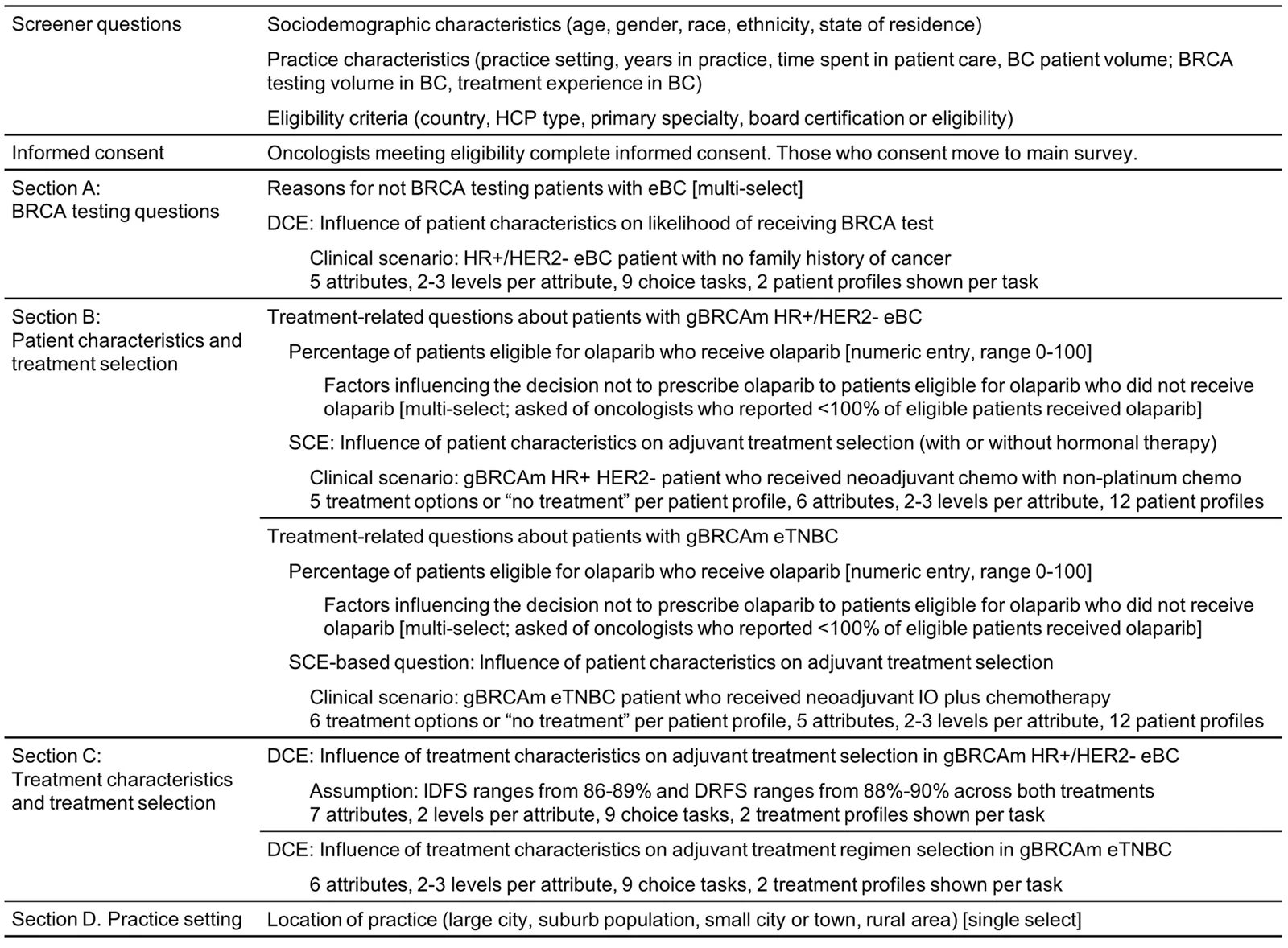
Quantitative survey flow diagram. BC, breast cancer; BRCA, breast cancer gene; DCE, discrete choice experiment; DRFS, distant relapse-free survival; eBC, early breast cancer; eTNBC, early triple-negative breast cancer; gBRCAm, germline BRCA mutation; HER2-, Human Epidermal Growth Factor Receptor 2-negative; HR+, hormone receptor-positive; IDFS, invasive disease-free survival; SCE, situational choice experiment.

The survey flow is depicted in **Figure 1**. Respondents first completed a survey screener, which contained questions to confirm eligible criteria and track quotas. Respondents who met eligibility criteria were then directed to give informed consent. Oncologists who gave informed consent proceeded to the survey. The survey was then divided into four sections. Section A contained questions on BRCA testing – in this section, the reasons for not testing an eBC patient for BRCA were captured via a multi-select response question. The oncologists completed the first DCE that evaluated the influence of patient characteristics on gBRCA testing decisions in a patient with HR+/HER2- eBC and no family history of breast cancer. Section B contained questions on patient characteristics and treatment selection – this section was divided into two additional sections. The first section was restricted to the gBRCAm HR+/HER2- eBC patient setting, and the second section was restricted to the gBRCAm eTNBC patient setting. The direct survey questions on the percentage of their olaparib-eligible patients with gBRCAm eBC who received olaparib and the factors influencing the decision not to prescribe olaparib to patients eligible for olaparib were asked in this section. Each sub-section ended with the SCE-based question used to characterize how patient characteristics influence treatment choice in the adjuvant setting for patients with gBRCAm eBC. Section C included the two DCEs evaluating the influence of treatment attributes on adjuvant treatment selection for patients with gBRCAm HR+/HER2- eBC and eTNBC, respectively. Section D included one additional direct survey question about the locale of oncologists’ primary practice (city, suburb, town, or rural).

#### 2.2.3 Development and validation of survey

Content for the survey, including the final selection of attributes and levels included in the DCEs, was informed by a literature review of the neoadjuvant and adjuvant treatments for HER2- BC and qualitative concept elicitation interviews with 16 oncologists (8 community-based and 8 academic-based) that occurred in October 2023 to evaluate how physicians view patient and treatment pathway characteristics. The attributes and levels were further refined through medical expert review to ensure clinical relevance across HER2- eBC subtypes. Levels for each attribute in the treatment selection DCEs represented the range of performance observed among the treatments available for the adjuvant treatment of high-risk HR+/HER2- eBC and eTNBC at the time of study conduct (29–40). Treatment regimens selected for inclusion in the SCE-based questions were based on guideline recommendations, the 2023 St. Gallen consensus statement, and qualitative concept elicitation interviews to capture common clinical practices in this rapidly evolving treatment landscape, including those not yet supported by evidence (3, 41, 42). The final survey instrument was pilot-tested via cognitive interviews with 8 oncologists (5 community-based and 3 academic-based) to test the content validity, ensure clarity of the questionnaire, and confirm and refine the attributes and levels included in the DCEs. The survey was designed to be completed in approximately 25 minutes, as shorter questionnaires have been reported to improve non-response bias (43).

### 2.3 Statistical analysis

Data analyses were conducted using IBM SPSS Statistics (RRID: SCR_016479) version 23.0 or higher for the descriptive and bivariate analyses, Sawtooth Software Lighthouse Studio version 9.15.4 or higher for the DCE, and R Project for Statistical Computing (RRID: SCR_001905) version 4.4.0 or higher for the analyses of the SCE-based questions.

#### 2.3.1 Direct survey questions

Descriptive statistics were used to characterize the oncologist sample, including sociodemographic characteristics and clinical practice characteristics. For categorical variables, frequencies and percentages were reported. For continuous or discrete variables, means and standard deviations were reported. Medians, minimums, and maximums were also reported for patient load characteristics and the number of patients referred for BRCA testing. Additionally, descriptive statistics, specifically percentages, were used to summarize the direct survey questions assessing the reasons for not testing an eBC patient for BRCA mutations and the factors influencing the decision not to prescribe olaparib to eligible patients with gBRCAm HER2- eBC. Means with standard deviations (SD) were used to report the percentage of gBRCAm HER2- eBC patients who are eligible for olaparib who receive olaparib.

#### 2.3.2 Discrete choice experiments

The DCE analyses were conducted per the recommendations of the ISPOR Good Research Practices for Conjoint Analysis Task Force (23). DCE data were analyzed using a Hierarchical Bayesian (HB) model, which generated parameter estimates (preference weights) for the factors or attributes included in the DCE exercise. The HB choice-probability model was based on conditional logit analysis, with effects coding applied to the attribute levels. These results were used to construct the joint posterior distribution of preference weights for the entire sample, providing both the mean and standard deviation for each attribute level. The preference weights indicate relative preference, meaning that only the differences between attribute-level estimates and the relative magnitude of those differences across attributes are interpretable. Using these weights, we can assess the magnitude of the trade-offs that physicians are willing to make among different attribute levels.

The parameter estimates (utilities) from the HB analysis enable the calculation of the conditional relative importance of each attribute. The analyses allowed us to examine the independent contribution of each patient characteristic attribute level to the treatment choice, while controlling for the effects of all other variables in the model. Relative importance estimates are ratio-scaled; hence, an attribute with a relative importance of 20 is twice as important as an attribute with a relative importance of 10.

#### 2.3.3 Situational choice experiment-based questions

For the SCE-based questions, the overall rate for selecting a treatment regardless of the levels shown was computed. In addition, the rate of selecting a treatment for a specific level was computed. The relative importance (RI) of each characteristic was measured by calculating the difference between the highest and lowest rates of treatment selection within an attribute, then the scores are scaled to sum to 100. The analyses allowed us to examine the independent contribution of each patient characteristic attribute level to the treatment choice, while controlling for the effects of all other variables in the model. This provided insight into how influential each characteristic was in treatment decisions. Higher scores indicate that the change within a patient characteristic had greater influence on choosing the selected treatment. Since the RIs were computed on aggregated rates, the RIs were descriptive and there were no *P*-tests for comparing which RIs were statistically different.

#### 2.3.4 Subgroup analyses

Select results, including the DCEs and SCE-based questions, were stratified by main practice setting. Bivariate analysis was performed to explore whether the RIs of patient characteristics in gBRCA testing decisions and the RIs of treatment attributes in adjuvant treatment selection differed by main practice setting, using independent samples t-tests. Power analysis was conducted using G*Power (RRID: SCR_013726) (44); this analysis assumed a two-tailed test, a Type 1 error rate of 0.05, and 80% statistical power to detect group differences. The target of 50 academic physicians and 100 community physicians allowed for the detection of a medium-sized effect (Cohen’s d = 0.49) between groups.

## 3 Results

**Figure 2:**
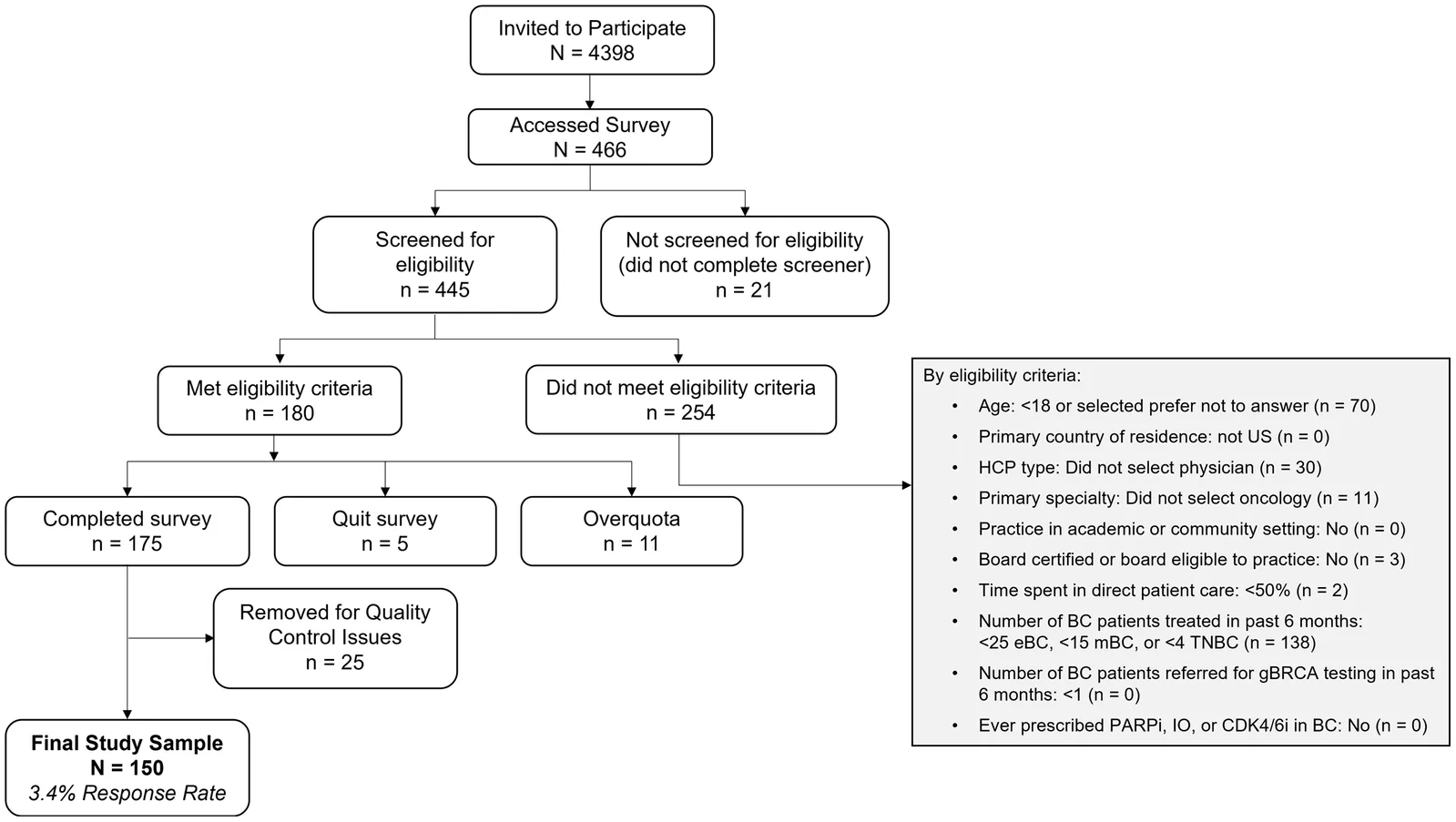
Participant recruitment flow diagram. BC, breast cancer; CDK4/6i, Cyclin-Dependent Kinases 4 and 6 inhibitor; eBC, early breast cancer; gBRCA, germline breast cancer gene; HCP, healthcare provider; IO, immunotherapy; mBC, metastatic breast cancer; PARPi, poly (ADP-ribose) polymerase (PARP) inhibitors; TNBC, triple negative breast cancer.

Data collection occurred from April to July 2024, and the median time to complete the survey was 24 minutes. The participant recruitment flow diagram is shown in **Figure 2**. The final analytic sample included 150 oncologists (55 academic, 95 community) with a mean age of 50.5 years (SD = 8.6) and 16.9 years (SD = 6.8) in practice post-fellowship. The majority of oncologists identified as male (75.3%, n = 113), while 22.0% (n = 33) identified as female. Almost two-thirds of the sample identified as White (64.7%, n = 97), 23.3% (n = 35) as Asian, 4.0% (n = 6) as Black or African American, and 10.0% (n = 15) as Hispanic ethnicity. Respondents were distributed across the four U.S. Census regions as follows: Northeast (20.1%, n = 30), Midwest (23.5%, n = 35), South (32.2%, n = 48), and West (24.2%, n = 36). Additional physician demographic and practice characteristics are shown in **Supplementary Table 2**.

### 3.1 DCE results: influence of patient characteristics on gBRCA testing at diagnosis in patients with HR+/HER2- eBC with no family history of breast cancer

**Figure 3:**
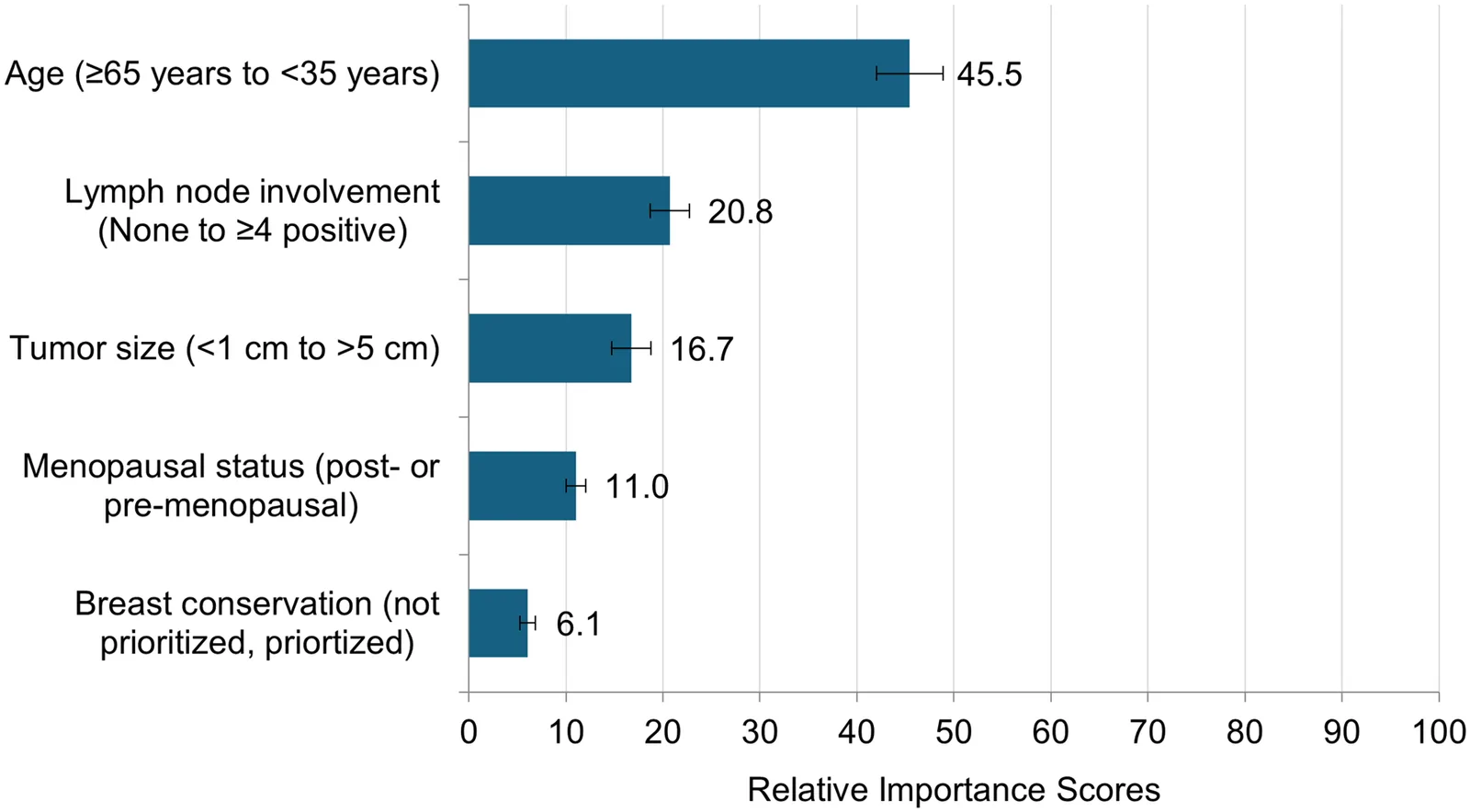
DCE results: patient characteristics influencing BRCA testing for HR+/HER2- eBC diagnosis, relative importance scores. Physicians were prompted in the DCE with: Which HR+ HER2- patient should be more likely to receive a BRCA test at diagnosis? Please assume that these patients have no family history of breast cancer. Relative importance scores sum to 100. Attribute levels are presented in parentheses. BRCA, breast cancer gene; DCE, discrete choice experiment; eBC, early breast cancer; HER2-, Human Epidermal Growth Factor Receptor 2-negative; HR+, hormone receptor-positive.

Age was the most important patient characteristic influencing oncologists to BRCA test newly-diagnosed patients with HR+/HER2- eBC without a family history of the disease (RI = 45.5), with oncologists more likely to test patients <65 years than those ≥65 years (**Figure 3**). Following in importance were lymph node involvement (RI = 20.8), tumor size (RI = 16.7), menopausal status (RI = 11.0), and breast conservation (RI = 6.1) (**Figure 3**). Relative importance of attributes did not differ by main practice setting (data not shown).

### 3.2 Barriers to gBRCA testing

**Figure 4:**
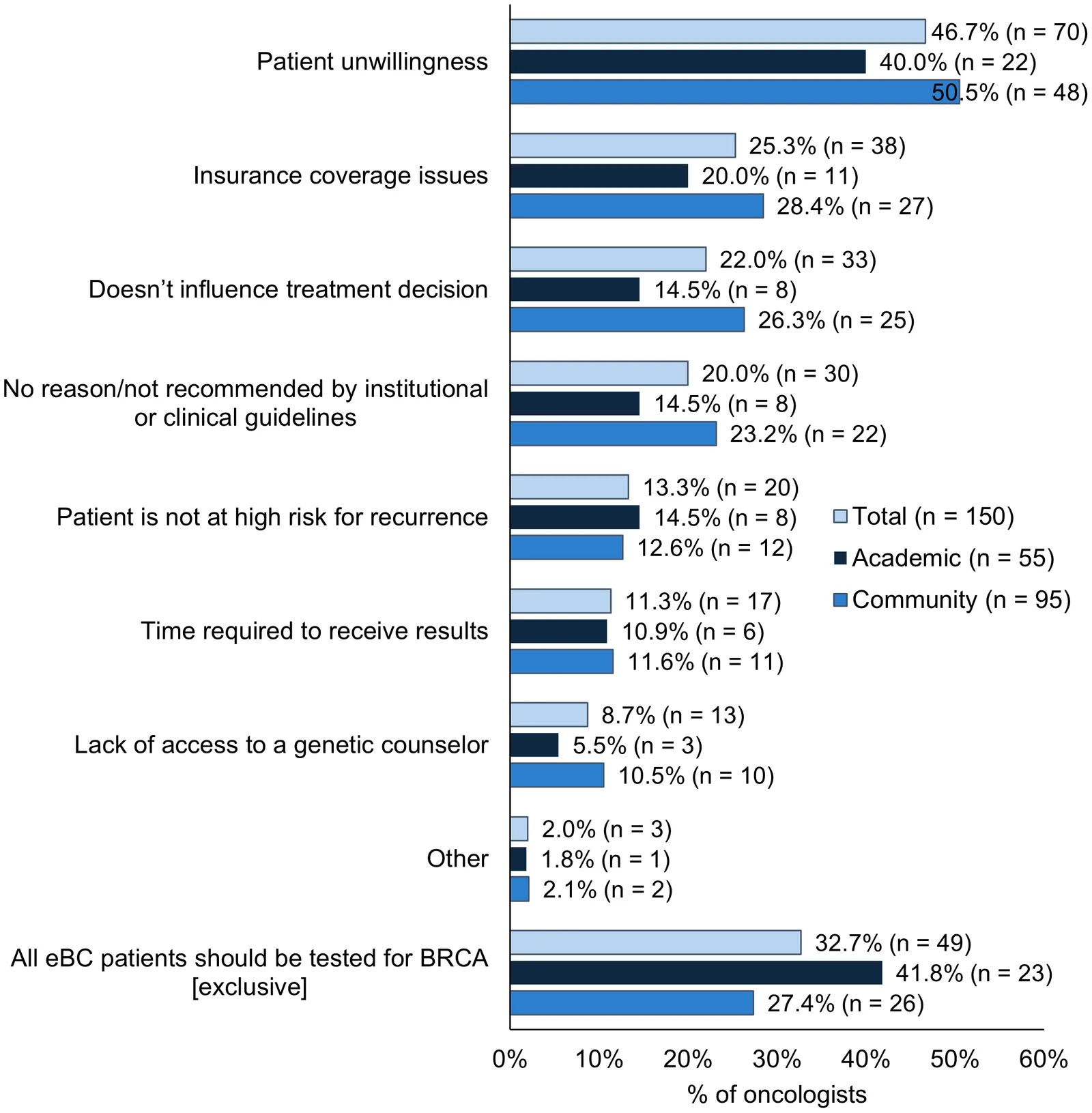
Barriers to testing patients with eBC for BRCA mutations. Physicians were asked: Which of the following are reasons for not testing an eBC patient for BRCA? Physicians were allowed to select multiple options (apart from “all eBC patients should be tested for BRCA”). BRCA, breast cancer gene; eBC, early breast cancer.

A total of 27.4% (26/95) of community oncologists and 41.8% (23/55) of academic oncologists reported that all patients with eBC should be tested for BRCA mutations. The oncologist-reported reasons for not gBRCA testing a patient with HER2- eBC are shown in **Figure 4**. Patient unwillingness was the most common reason cited by oncologists for not conducting gBRCA testing, reported by 50.5% (48/95) of community oncologists and 40.0% (22/55) of academic oncologists. Notably, 26.3% (25/95) of community oncologists and 14.5% (8/55) of academic oncologists reported that gBRCA testing results do not influence their treatment decisions for eBC.

### 3.3 Patient characteristics guiding adjuvant treatment decisions

Olaparib-containing regimens were the preferred treatment choice among oncologists for patients with gBRCAm HR+/HER2- eBC or eTNBC (83.9% [1511/1800] and 83.7% [1507/1800], respectively; **Table 2**). The most influential patient characteristics guiding adjuvant treatment decisions differed by disease subtype.

**Table 2:** Results for SCE-based questions: proportion of time an adjuvant treatment was chosen across all tasks.

Adjuvant treatment	Total (n = 150)	Academic (n = 55)	Community (n = 95)
gBRCAm HR+/HER2- eBC, n (%)			
Olaparib only	343 (19.1)	75 (11.4)	268 (23.5)
CDK4/6i only	177 (9.8)	58 (8.8)	119 (10.4)
Olaparib + CDK4/6i	538 (29.9)	224 (33.9)	314 (27.5)
Olaparib followed by CDK4/6i	351 (19.5)	143 (21.7)	208 (18.2)
CDK4/6i followed by olaparib	279 (15.5)	119 (18.0)	160 (14.0)
No treatment	112 (6.2)	41 (6.2)	71 (6.2)
gBRCAm eTNBC, n (%)			
Olaparib only	331 (18.4)	99 (15.0)	232 (20.4)
Olaparib + IO	575 (31.9)	229 (34.7)	346 (30.4)
IO only	188 (10.4)	63 (9.5)	125 (11.0)
Olaparib followed by IO	213 (11.8)	84 (12.7)	129 (11.3)
IO followed by olaparib	388 (21.6)	155 (23.5)	233 (20.4)
Capecitabine only	75 (4.2)	19 (2.9)	56 (4.9)
No treatment	30 (1.7)	11 (1.7)	19 (1.7)

The denominator for calculating the proportion of time an adjuvant treatment was chosen across all tasks was 1800 (150 respondents x 12 choice tasks = 1800 choice tasks).

CDK4/6i, Cyclin-Dependent Kinases 4 and 6 inhibitor; eBC, early breast cancer; eTNBC, early triple-negative breast cancer; gBRCAm, germline BRCA mutation; HER2-, Human Epidermal Growth Factor Receptor 2-negative; HR+, hormone receptor-positive; IO, immunotherapy; SCE, situational choice experiment.

For patients with gBRCAm HR+/HER2- eBC, lymph node involvement was most influential in selecting olaparib monotherapy or an olaparib-containing regimen (**Table 3**). While lymph node status was the most important factor in selecting olaparib monotherapy for both practice settings, it was more than twice as influential for community oncologists (RI = 57.1) compared to their academic counterparts (RI = 27.3) (**Supplementary Table 3**).

**Table 3:** Results for SCE-based questions: relative importance scores for patient characteristics in treatment selection for HER2- eBC.

gBRCAm HR+/HER2- eBC*		Relative importance scores (sum to 100 for each treatment)					
Attribute	Olaparib only	CDK4/6i only	Olaparib + CDK4/6i	Olaparib followed by CDK4/6i	CDK4/6i followed by olaparib	No treatment	
Age (45 years, 56 years)	4.6	4.6	1.7	0.9	4.4	1.4	
Stage (IIB, III)	0.7	8.3	0.9	18.7	13.1	4.3	
Ki-67 score (low, intermediate, high)	20.7	20.8	19.9	22.7	10.9	18.3	
pCR status (pCR, no pCR)	0.9	36.2	22.0	18.4	12.7	28.7	
Lymph node involvement (None, 3 or less, ≥4)	62.0	23.6	39.2	34.8	54.6	38.7	
Oncotype Dx score (26, 50)	11.2	6.5	16.3	4.5	4.4	8.6	
gBRCAm eTNBC^†^							
**Attribute**	**Olaparib only**	**Olaparib + IO**	**IO only**	**Olaparib** **followed by IO**	**IO followed by olaparib**	**Capecitabine only**	**No treatment**
Age (45 years, 56 years)	0.9	8.3	12.5	44.2	43.6	5.0	4.1
Stage (IIB, III)	11.9	4.5	10.4	2.1	4.0	11.8	12.2
Ki-67 score (low, intermediate, high)	30.1	19.2	3.1	9.5	8.9	12.6	3.1
pCR status (pCR, no pCR)	39.3	46.8	70.8	18.9	31.7	65.5	53.1
Lymph node involvement (pN0 < pT2, pN0 ≥ pT2, ≥ pN1)	17.8	21.2	3.1	25.3	11.9	5.0	27.6

Relative importance scores sum to 100 for each treatment. Relative importance scores are interpreted as the percentage of total importance (difference between most influential versus least influential level of the attribute) within each treatment choice. Higher scores indicate that the change within a patient characteristic had greater influence on choosing the selected treatment. Relative importance scores are ratio-scaled; hence, an attribute with a relative importance of 20 is twice as important as an attribute with a relative importance of 10. Attribute levels are presented in parentheses.

*Physicians were prompted with: What are you most likely to prescribe as a treatment in the adjuvant setting with or without hormonal therapy for a gBRCAm HR+ HER2- patient with the following characteristics? Please assume the patient received neoadjuvant chemo with non-platinum chemo.

^†^ Physicians were prompted with What are you most likely to prescribe as adjuvant therapy for a gBRCAm TNBC patient with the following characteristics? Please assume the patient received neoadjuvant IO plus chemotherapy.

CDK4/6i, Cyclin-Dependent Kinases 4 and 6 inhibitor; eBC, early breast cancer; eTNBC, early triple-negative breast cancer; gBRCAm, germline BRCA mutation; HER2-, Human Epidermal Growth Factor Receptor 2-negative; HR+, hormone receptor-positive; IO, immunotherapy; pCR, pathological complete response; SCE, situational choice experiment.

For patients with gBRCAm eTNBC, pathological complete response (pCR) status was most influential for selecting olaparib monotherapy and olaparib combined with IO; age was most influential in selecting olaparib followed by IO and IO followed by olaparib regimens (**Table 3**). Academic and community oncologists differed in their approach to selecting olaparib monotherapy, with academic oncologists focusing on Ki-67 score and community oncologists placing greater emphasis on pCR status (**Supplementary Table 3**).

### 3.4 DCE results: influence of treatment characteristics on treatment selection

**Figure 5:**
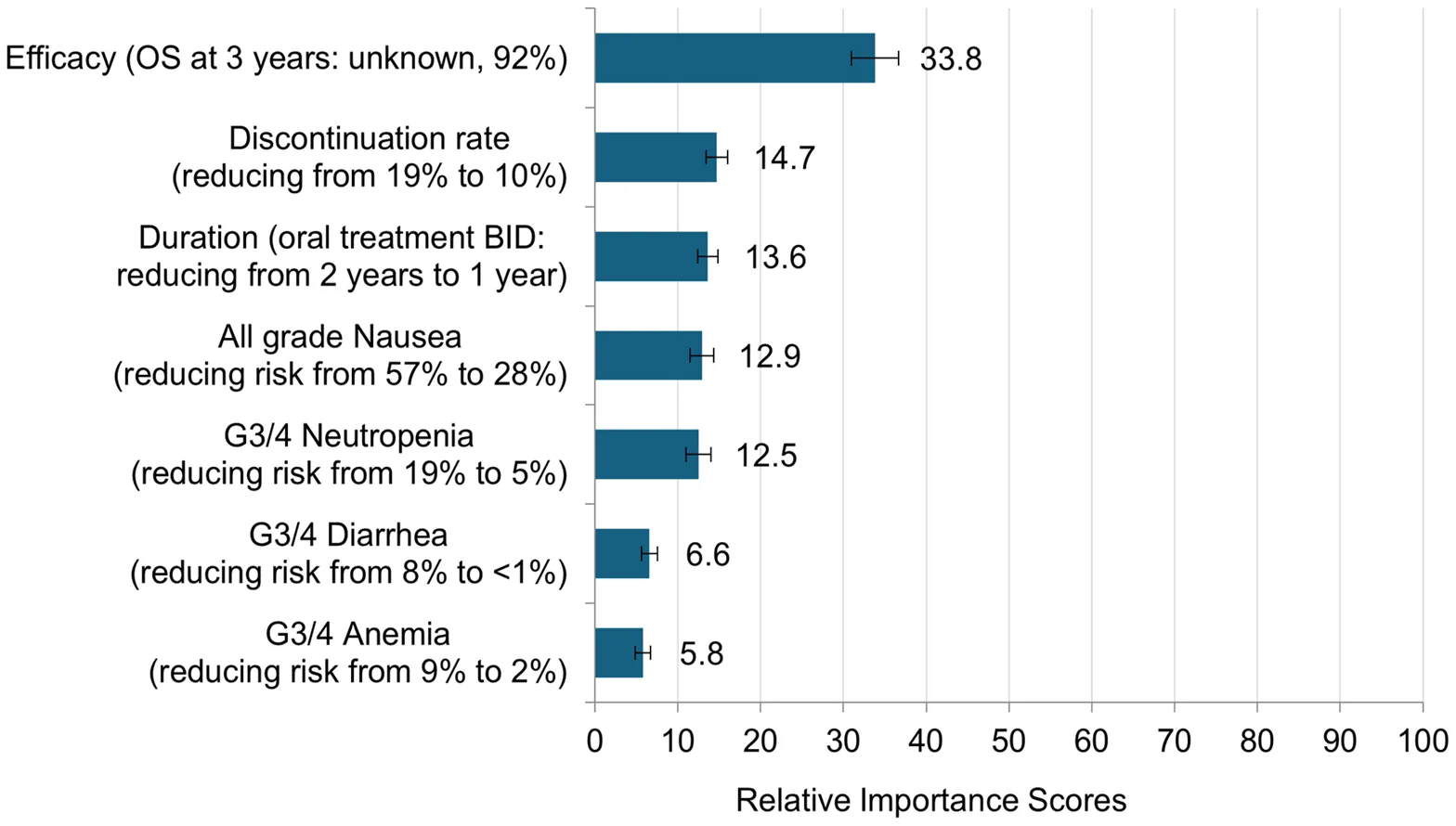
DCE results: treatment characteristics influencing treatment selection for gBRCAm HR+/HER2- eBC, relative importance estimates. Physicians were prompted in the DCE with: Which adjuvant treatment would you be more likely to prescribe to a BRCAm patient with HR+ HER2- eBC? Please assume that IDFS ranges from 86-89% and DRFS ranges from 88%-90% across both treatments. Relative importance scores sum to 100. Attribute levels are presented in parentheses. BID, twice a day; DCE, discrete choice experiment; DRFS, distant relapse-free survival; eBC, early breast cancer; G3/4; grade 3/4; gBRCAm, germline BRCA mutation; HER2-, Human Epidermal Growth Factor Receptor 2-negative; HR+, hormone receptor-positive; IDFS, invasive disease-free survival; OS, overall survival.

**Figure 6:**
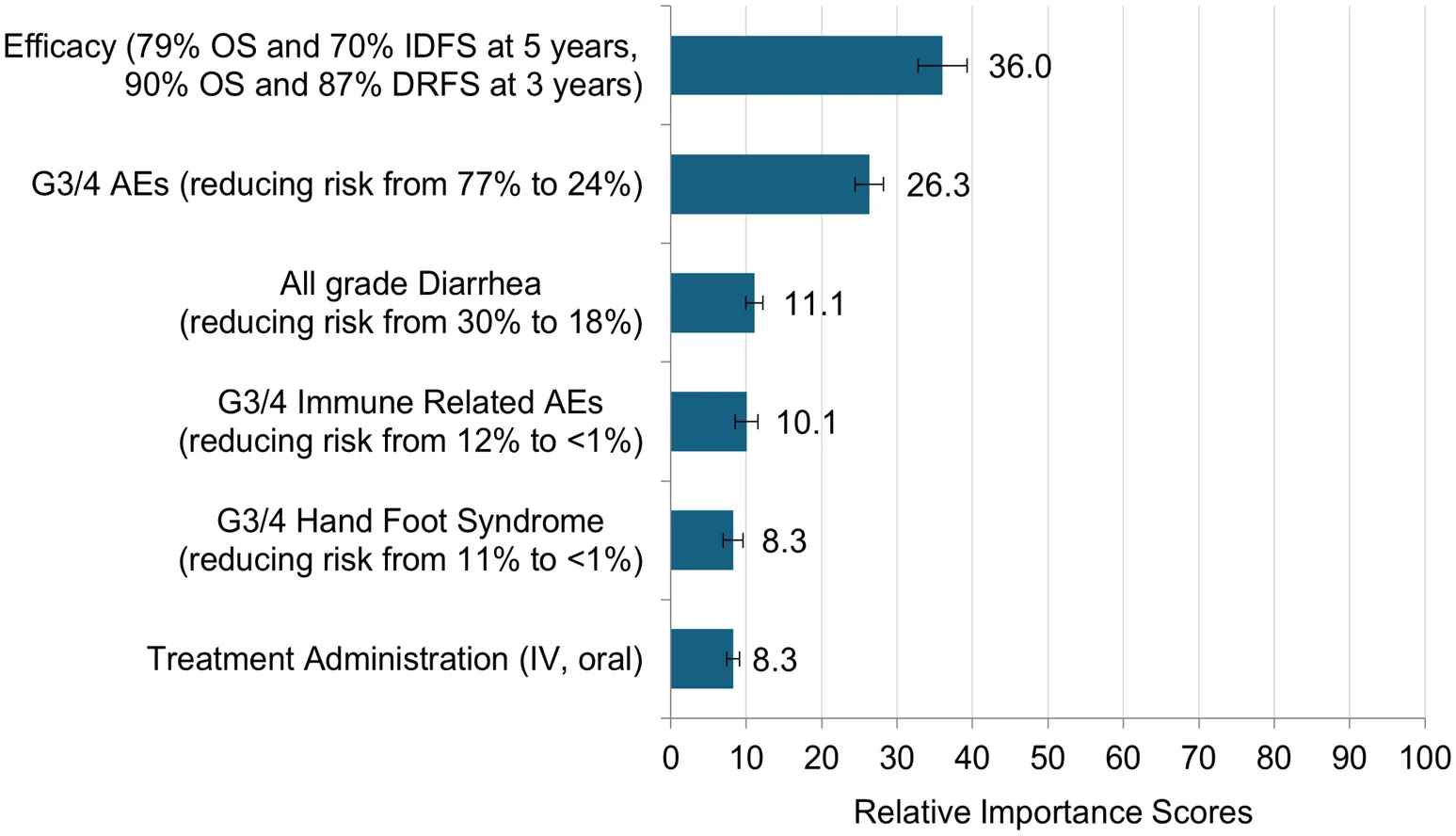
DCE results: treatment characteristics influencing treatment selection for gBRCAm TNBC in eBC, relative importance estimates. Physicians were prompted in the DCE with: Which treatment regimen would you be more likely to prescribe to a BRCAm patient with TNBC in early breast cancer? Relative importance scores sum to 100. Attribute levels are presented in parentheses. AEs, adverse events; DCE, discrete choice experiment; DRFS, distant relapse-free survival; eBC, early breast cancer; G3/4; grade 3/4; gBRCAm, germline BRCA mutation; HER2-, Human Epidermal Growth Factor Receptor 2-negative; HR+, hormone receptor-positive; IDFS, invasive disease-free survival; IV, intravenous; OS, overall survival; TNBC, triple negative breast cancer.

In patients with gBRCAm HR+/HER2- eBC, efficacy was the most important attribute driving adjuvant treatment selection and was more than twice as important as discontinuation rate and duration of treatment (**Figure 5**). Efficacy and risk of grade 3/4 adverse events were the most important attributes driving adjuvant treatment selection for patients with gBRCAm eTNBC, which were more than twice as important as all other attributes (**Figure 6**). Preferences for adjuvant treatment did not differ by main practice setting for either HER2- BC subtype.

### 3.5 Barriers to adjuvant olaparib for treating gBRCAm HER2- eBC

On average, oncologists reported that 68.8% (SD = 26.8) of their olaparib-eligible patients with gBRCAm HR+/HER2- eBC and 72.3% (SD = 27.0) of their olaparib-eligible patients with gBRCAm eTNBC received olaparib (data not shown). These results indicate that, on average, approximately 30% (HR+/HER2-: 100 – 68.8 = 31.2, eTNBC: 100 – 72.3 = 27.7) of eligible patients did not receive olaparib, according to surveyed oncologists (data not shown).

**Figure 7:**
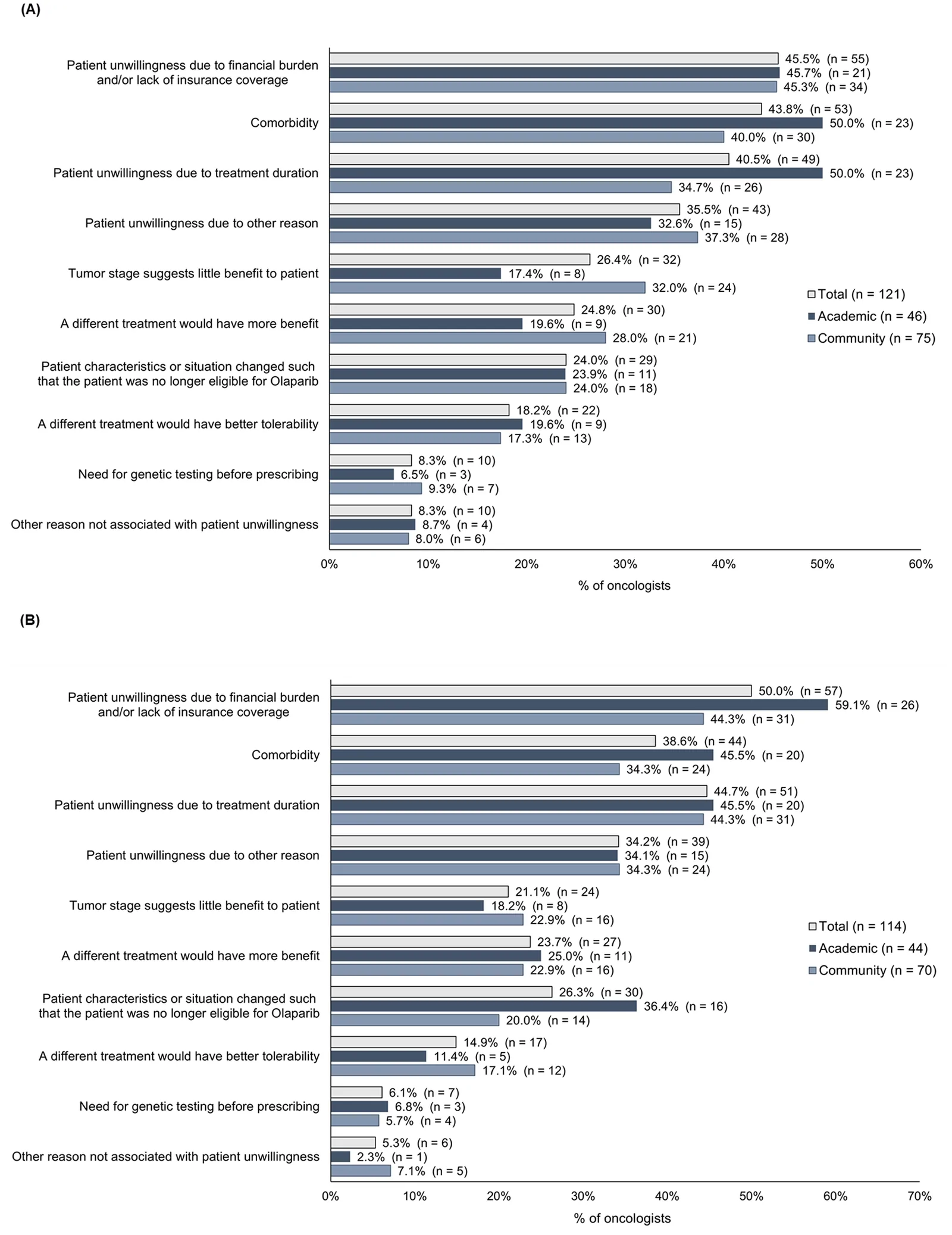
Barriers to prescribing adjuvant olaparib in HER2- eBC: total oncologists and by main practice setting. **(A)** Patients with gBRCAm HR+/HER2- eBC. **(B)** Patients with gBRCAm eTNBC. Oncologists were asked this question twice, once for gBRCAm HR+ HER2- eBC patients and again for gBRCAm TNBC patients. Question wording: For your [gBRCAm HR+ HER2- eBC patients/gBRCAm TNBC patients] who are otherwise eligible for Olaparib and did not receive Olaparib, which of the following factors influenced the decision to not prescribe Olaparib? Only asked of oncologists who reported <100% of their gBRCAm patients eligible for olaparib received olaparib (HR+/HER2- eBC: n = 121; eTNBC: n = 114). eBC, early breast cancer; eTNBC, early triple-negative breast cancer; gBRCAm, germline BRCA mutation; HER2-, Human Epidermal Growth Factor Receptor 2-negative; HR+, hormone receptor-positive; TNBC, triple-negative breast cancer.

Patient unwillingness to receive adjuvant olaparib due to financial burden or lack of insurance coverage (HR+/HER2-: 45.5% [55/121]; TNBC: 50.0%, [57/114]), treatment duration (HR+/HER2-: 40.5% [49/121]; TNBC: 44.7%, [51/114]), or other unspecified reasons (HR+/HER2-: 35.5% [43/121]; TNBC: 34.2%, [39/114]) was commonly reported by the surveyed oncologists as a barrier in both patient settings. Presence of a comorbidity was another barrier to prescribing adjuvant olaparib (HR+/HER2-: 43.8% [53/121]; TNBC: 38.6%, [44/114]), reported by oncologists (**Figure 7**).

## 4 Discussion

Since the initiation of the OlympiA trial, which assessed the efficacy of olaparib in the adjuvant setting, several new agents have become available, including pembrolizumab, abemaciclib, and ribociclib (16–18). Their availability has complicated the integration of olaparib in the high-risk gBRCAm HER2- eBC adjuvant setting, as there is limited evidence to guide the selection, sequencing, or combination of these new therapies (20). As a result, understanding how oncologists make decisions regarding adjuvant treatment for HER2- eBC has become increasingly important.

To our knowledge, no research has examined physician preferences or the factors influencing their decisions regarding gBRCA testing or the selection of adjuvant therapy within the current treatment landscape for gBRCAm HER2- eBC. Therefore, this study aimed to identify factors influencing oncologists’ decisions for testing and treatment in this population. Our study found that patient age was the primary factor in choosing to BRCA test a HR+/HER2- eBC patient with no family history of cancer, with patients <65 years old more likely to receive a BRCA test, despite the National Comprehensive Cancer Network® (NCCN®) Clinical Practice Guidelines in Oncology (NCCN Guidelines®) recommendation to test patients of any age to determine eligibility for olaparib (5). The American Society of Clinical Oncology (ASCO) and the Society of Surgical Oncology (SSO) have issued joint guidelines recommending that *BRCA1/2* mutation testing should be offered to all newly diagnosed patients with breast cancer ≤65 years and select patients >65 years based on personal history, family history, ancestry, or eligibility for PARPi therapy (45). Additionally, guidelines from the American Society of Breast Surgeons (ASBrS) recommend gBRCA testing for all patients with a personal history of breast cancer, regardless of age (46). The majority of oncologists indicated a willingness to prescribe adjuvant olaparib for high-risk, gBRCAm HER2- eBC. Factors most influential in driving treatment choice were lymph node involvement for HR+/HER2- eBC when selecting olaparib-based regimens and pCR status for eTNBC when selecting olaparib monotherapy and olaparib combined with IO. Additionally, patient age was the most influential factor in the decision to prescribe either olaparib followed by IO or IO followed by olaparib in patients with eTNBC.

### 4.1 gBRCA testing decisions, attitudes, and behaviors

Age emerged as the most influential factor guiding oncologists’ decisions to gBRCA test patients with HR+/HER2- eBC who do not have a family history of BC, with patients <65 years being more likely to receive a BRCA test than those ≥65 years. This finding aligns with the ASCO-SSO clinical practice guidelines’ recommendation to offer *BRCA1/2* mutation testing to all patients 65 years or younger at diagnosis (45) and the NCCN Guidelines'^®^ recommendation to test all patients 50 years or younger at diagnosis for risk assessment (5). However, guidelines also recommend gBRCA testing for all patients with high-risk, HER2- eBC regardless of age, as defined by the phase III OlympiA trial (30), to determine eligibility for PARPi therapy (3, 5, 42, 45). In this study, patient age was twice as important as tumor size and lymph node involvement, factors used to assess high-risk status (3, 30, 42), in decisions to order gBRCA testing. This discrepancy may suggest a need for educational initiatives to raise physician awareness of guidelines. However, the interpretation of this finding may be limited by the survey design, as physicians were not asked to clarify whether BRCA testing was intended for risk assessment or to guide treatment decisions. As a result, responses may have varied based on how individual physicians interpreted the question.

Approximately one-third of surveyed oncologists reported that all patients with eBC should be tested for BRCA mutations, which reflects trends from a 2023 Association of Cancer Care Centers (ACCC) survey reporting 23% (26/115) of providers routinely ordered gBRCA testing for all patients with eBC—a three-fold increase from 7% (7/95) in 2018 (47). This may be due to the impact of updated clinical guidelines and the approval of adjuvant olaparib in 2022, which requires gBRCA testing to determine eligibility (3, 5, 42, 45). However, nearly one-fourth of oncologists reported that gBRCA testing does not influence their treatment decisions. Community oncologists were more likely to report this than academic counterparts, who were more inclined to test all patients with eBC (42% academic oncologists vs 27% community oncologists). While qualitative research is needed to better understand these differences, our findings suggest a need for targeted educational efforts in the community setting to emphasize the clinical rationale of gBRCA testing in patients with eBC.

### 4.2 Preferred adjuvant therapy for gBRCAm HER2- eBC

Olaparib-based regimens were the preferred treatment choice for adjuvant therapy for gBRCAm HER2- eBC, highlighting the important role PARPi play in the current landscape. However, education gaps may still exist regarding the use of PARPi in the adjuvant setting and the eligibility criteria for this therapy, as evidenced by the nearly a quarter of surveyed oncologists who reported that BRCA testing did not influence their treatment decisions and that oncologists reported that approximately 30%, on average, of their patients who were eligible for olaparib did not receive the medication for a variety of reasons, including patient refusal.

Interestingly, approximately 65% of oncologists selected an olaparib combination or olaparib sequential therapy for both gBRCAm HR+/HER2- eBC (olaparib combined or sequenced with CDK4/6i) and gBRCAm eTNBC (post-neoadjuvant olaparib combined or sequenced with IO). Moreover, an olaparib combination therapy was chosen more often than guideline-supported monotherapy in both gBRCAm HR+/HER2- eBC (olaparib combined with CDK4/6i, 29.9%) and gBRCAm eTNBC (olaparib combined with IO, 31.9%). Currently, the option to combine or sequence olaparib and a CDK4/6i for eligible patients remains unsupported by efficacy or safety data, especially combination olaparib + CDK4/6i due to overlapping toxicity (42). However, the NCCN Guidelines, ESMO guidelines, and the 2023 St. Gallen consensus do recognize the sequential approach (3, 41, 42), with the latter two specifying olaparib should come first (41, 42). Likewise, while the tolerability of combining immunotherapy and PARPi has been demonstrated for metastatic TNBC (20, 48–50), the clinical benefit and tolerability of combining PARPi and IO in eBC has not been established. Nonetheless, NCCN Guidelines support consideration of adjuvant pembrolizumab for patients with high-risk gBRCAm TNBC who received a pembrolizumab-containing regimen preoperatively and have residual disease (3), ESMO guidelines support the combination of immune checkpoint inhibitors and olaparib on an individual basis in patients with gBRCAm and high-risk TNBC (42), and the 2023 St. Gallen consensus supports adjuvant pembrolizumab combined with olaparib in patients with TNBC and *BRCA1* mutation who received neoadjuvant KEYNOTE-522 treatment at surgery and have residual disease (41). While some physicians are adopting emerging treatment strategies with limited evidence, community oncologists favored olaparib monotherapy more than twice as often as academic oncologists. These findings suggest that community physicians may adhere more closely to guideline recommendations with stronger supporting evidence, while academic oncologists may be more willing to exercise clinical discretion.

### 4.3 Patient characteristics influencing treatment decisions

The patient characteristics influencing olaparib-based adjuvant treatment selection in our study closely aligned with guideline recommendations (3, 42). Lymph node involvement was the most influential patient characteristic for choosing adjuvant olaparib monotherapy, combination, or sequential therapy for gBRCAm HR+/HER2- eBC. Among patients with gBRCAm eTNBC, pCR status was the most common factor for selecting olaparib monotherapy or combination therapy. Age was most influential in the selection of olaparib sequential therapy, as age may affect the tolerance of certain treatments.

### 4.4 Treatment attributes influencing treatment decisions

When selecting a treatment for gBRCAm HR+/HER2- eBC, efficacy was the most important treatment attribute—more than twice as important as discontinuation rate and duration of treatment. Furthermore, efficacy and risk of grade 3/4 adverse events were the most important treatment attributes driving adjuvant treatment selection for gBRCAm eTNBC—more than twice as important as all other attributes. These results emphasize the role of efficacy in physicians’ treatment decisions for gBRCAm HER2- eBC, regardless of practice setting. Notably, olaparib is the only targeted therapy for gBRCAm HER2- eBC that has demonstrated a significant overall survival (OS) benefit (20, 51, 52).

### 4.5 Barriers to receiving olaparib

Physicians reported that approximately 30%, on average, of their eligible patients did not receive olaparib, most commonly due to patient unwillingness related to financial burden, treatment duration, or other unspecified reasons. However, the survey did not clarify whether the concern about treatment duration stemmed from the 1-year duration of olaparib itself or from the burden of receiving any additional treatment (3). This lack of specificity may have contributed to variation in responses. Other adjuvant treatments for HER2- eBC often require longer durations of treatment. For example, adjuvant endocrine therapy is typically prescribed for 5 to 10 years in patients with HR-positive disease, and adjuvant CDK4/6i is administered for 2 to 3 years concurrently with endocrine therapy (3). Compared to these regimens, the 1-year treatment duration for olaparib is comparatively modest, suggesting that the perceived burden may be driven more by cumulative treatment expectations than by olaparib alone (3). However, the cumulative burden of multiple therapies, both in terms of side effect management and financial impact, may still influence patients’ willingness to begin additional treatment (53). Therefore, these barriers should be addressed in patient care discussions. Additionally, further investigation should compare factors impacting patient preferences for adjuvant olaparib with other adjuvant therapies for HER2- eBC to better understand the barriers to adjuvant olaparib uptake.

### 4.6 Strengths and limitations

DCEs are commonly used to assess physician preferences and evaluate trade-offs in decision-making. With strong foundations in psychology and economics, DCEs are increasingly used in healthcare to facilitate shared decision-making. Their strengths include robust preference elicitation methods and adherence to ISPOR best practices (23). In contrast, SCEs are less common in real-world evidence and health outcomes research and are used in commercial market research. They are grounded in psychology and economics and share a similar design to DCEs, making ISPOR guidelines also applicable. Another strength of our study is the inclusion of cognitive testing prior to the formal implementation of the survey, which strengthens the methodological rigor and improves the reliability of the results. Additionally, the attributes and levels included in the DCE and SCE-based questions were refined through input from medical experts to ensure clinical relevance across HER2- BC subtypes.

The study also stands out in its contemporaneous evaluation of physician preferences for testing and treatment, ensuring relevance to the evolving eBC treatment landscape. The study captures how the rationale for gBRCA testing has become stronger following the findings of the OlympiA trial, reflecting a key shift in clinical practice. However, the study also identifies a gap in fully recognizing this shift, underscoring an opportunity for greater integration of recent trial findings into clinical decision-making. Additionally, the study reveals that healthcare professionals are still exploring the optimal treatment sequences for patients with gBRCAm. This insight emphasizes the need for continued research and clinical flexibility, providing valuable guidance for future studies and clinical practices.

The self-reported nature of the survey introduces potential response biases, such as inaccurate recall or false reporting, whether intentional or unintentional. Convenience sampling may also lead to overrepresentation of certain participants and healthcare settings (i.e., US), limiting the generalizability of results. As the study was conducted with oncologists practicing in the US, results are not generalizable to oncologists practicing outside of the US. Further, because oncologists were required to have a history of prescribing PARPi, IO, or CDK4/6i in BC and have seen ≥25 eBC cases in the past 6 months, the study may have overestimated guideline familiarity and olaparib uptake compared with a broader community oncology practice. It is possible that the awareness of guidelines may be even poorer among less-exposed oncologists.

In addition, the DCE methodology involved respondents choosing between hypothetical patient profiles with a set of attributes that, like patient preference research, may not reflect all factors that can influence choice. Similarly, the SCE-based questions involved respondents choosing treatment for hypothetical patients that may not capture all the aspects that would affect a real-world choice by the physician. Consequently, the study’s results may not fully reflect real-world decisions, which can be influenced by unknown factors not captured in the survey or qualitative research. The data collected in the DCEs and SCE-based questions are based on simulated clinical decisions but lack the true clinical, financial, or emotional consequences of real choices, leading to potential discrepancies between stated and actual preferences. To minimize hypothetical bias, choice questions that closely resemble realistic clinical scenarios and align with clinical evidence were constructed. Likewise, the treatment regimens included in the SCE-based questions may not reflect all treatment regimens oncologists would consider or prescribe given the patient profiles. However, we tested the SCE-based questions in cognitive interviews, so we can be relatively certain that we captured the ones most likely to be prescribed.

Lastly, this research only considers the perspective of clinicians; it may be beneficial for future research to incorporate the perspective of patients regarding gBRCA testing and adjuvant treatment selection decisions in HER2- eBC.

### 4.7 Conclusions

Our study demonstrates a disconnect between guideline recommendations and the current clinical practice for gBRCA testing in eBC. Although guidelines recommend gBRCA testing for all patients with BC to determine eligibility for adjuvant olaparib, age continues to drive testing decisions, and a notable proportion of oncologists reported that BRCA status does not inform their adjuvant treatment selection. Educational efforts are needed to improve awareness of the current guidelines and the clinical rationale for gBRCA testing. Improved integration of gBRCA testing into treatment decision-making is needed to ensure that olaparib-eligible patients are identified and receive the most effective, personalized therapy.

## Data Availability

The datasets presented in this article are not readily available because the participants of this study did not give written consent for their data to be shared publicly, so only deidentified aggregate data will be shared upon reasonable request. Requests to access the datasets should be directed to Qixin Li, sandy.li1@astrazeneca.com.

## Author disclaimer

Citation 3 is referenced with permission from the NCCN Clinical Practice Guidelines in Oncology (NCCN Guidelines®) for Breast Cancer V.4.2025. © National Comprehensive Cancer Network, Inc. 2025. All rights reserved. Accessed April 30, 2025. To view the most recent and complete version of the guideline, go online to NCCN.org. Citation 5 is referenced with permission from the NCCN Clinical Practice Guidelines in Oncology (NCCN Guidelines®) for Genetic/Familial High-Risk Assessment: Breast, Ovarian, Pancreatic, and Prostate V.2.2025. © National Comprehensive Cancer Network, Inc. 2025. All rights reserved. Accessed April 30, 2025. To view the most recent and complete version of the guideline, go online to NCCN.org. NCCN makes no warranties of any kind whatsoever regarding their content, use or application and disclaims any responsibility for their application or use in any way.
