# Supplementary material for: Factors influencing oncologists’ decisions for germline BRCA testing and adjuvant therapy selection in HER2-negative early breast cancer
Source: Front Oncol. 2026 Sep 3;16:1854965. doi: 10.3389/fonc.2026.1854965 (PMC13581792; doi:10.3389/fonc.2026.1854965)
Supplement: Supplementary file 1 [file SupplementaryFile1.pdf]

Supplementary Material

1 Supplementary Tables

Supplementary Table 1. Survey Questions

Variable	Question	Format
**Survey Screener - Sociodemographic characteristics**		
Age	What is your age?	Whole number (≥18 to proceed)
Gender	How would you describe your gender?	Female<br>Male<br>Other, such as non-binary, gender fluid, gender queer<br>Prefer not to answer
Race	How would you describe your race?	White<br>Black or African American<br>Asian<br>Native Hawaiian or other Pacific Islander<br>American Indian or Alaskan Native<br>Some other race or origin<br>Prefer not to answer
Ethnicity	How would you describe your ethnicity?	Hispanic or Latino<br>Not Hispanic or Latino<br>Prefer not to answer
Country	In which country do you **primarily** reside?	Canada<br>France<br>Germany<br>United Kingdom<br>United States [must select to qualify]<br>Other
State	In which state or territory do you **primarily** reside?<br>[coded into US Census Region]	DROP DOWN LIST [50 states + D.C. and Puerto Rico]<br>Prefer not to answer **[exclusive]**
**Survey Screener - Practice characteristics**		
Type of healthcare provider	Which type of healthcare provider are you?	Dietitian<br>Genetic Counselor<br>Nurse Practitioner<br>Physician Assistant/Associate<br>Surgeon<br>Physician [must select to qualify]<br>Other healthcare provider
Primary specialty	Which of the specialties below best describes your primary area of practice?	General Practice/Family Medicine<br>Internal Medicine<br>Obstetrics/Gynecology<br>Oncology [must select to qualify]<br>Endocrinology<br>Cardiology<br>Gastroenterology/Hepatology<br>Psychiatry<br>Other Specialty

Confidential- Oracle Internal\Including External Recipients

Variable	Question	Format
Primary practice setting	Which of the following specialties below best describes your primary area of practice?	Academic hospital center/cancer center<br>Specialized academic/teaching clinic<br>Non-academic hospital/cancer center<br>Community-based solo/group private practice<br>Non-academic/non-teaching oncology private clinic or group practice<br>Government/VA hospital [Terminate if selected]<br>Other, please specify [Terminate if selected]
Board certification or eligibility	Are you board certified or eligible to practice in your specialty?	Yes, Board Certified<br>Yes, Board Eligible<br>No [Terminate if selected]
Years of clinical practice experience	How many years of clinical practice experience do you have **after** completing your fellowship?	Whole number
Time spent in patient care	How much of your time do you spend in direct patient care in comparison to teaching, research, administrative work, etc?	Whole number (≥50 to proceed)
Number of breast cancer patients	How many patients with breast cancer did you see or treat in the **past 6 months**?	Whole number (eBC patients ≥ 25 to proceed; mBC patients ≥ 15 to proceed; TNBC patients ≥ 4 to proceed)
Breast cancer patients referred for testing	How many patients with breast cancer did you refer for BRCA testing in the **past 6 months**?	Whole number (≥ 1 to proceed)
Prescribed PARP inhibitor	Have you ever prescribed a PARP inhibitor such as Olaparib (Lynparza) or Talazoparib (Talzenna) to treat breast cancer?	Yes, in metastatic breast cancer only<br>Yes, in both metastatic and early breast cancer<br>Yes, in early breast cancer only<br>No, not in metastatic or early breast cancer
Prescribed immunotherapy	Have you ever prescribed an immunotherapy such as pembrolizumab (Keytruda) or atezolizumab (Tecentriq) to treat breast cancer?	Yes, in metastatic breast cancer only<br>Yes, in both metastatic and early breast cancer<br>Yes, in early breast cancer only<br>No, not in metastatic or early breast cancer
Prescribed a CDK4/6i	Have you ever prescribed a CDK4/6i such as abemaciclib (Verzenio), ribociclib (Kisqali), or palbociclib (Ibrance) to treat breast cancer?	Yes, in metastatic breast cancer only<br>Yes, in both metastatic and early breast cancer<br>Yes, in early breast cancer only<br>No, not in metastatic or early breast cancer
**Section A: BRCA Testing and Genetic Status**		
Reason for not testing	Which of the following are reasons for not testing an eBC patient for BRCA?<br><br>(Multi-select)	Patient unwillingness<br>No reason/not recommended by institutional or clinical guidelines<br>Time required to receive results<br>Doesn't influence treatment decision<br>Insurance coverage issues<br>Patient is not at high risk for recurrence<br>Lack of access to a genetic counselor<br>Other<br>All eBC patients should be tested for BRCA [**exclusive**]
DCE: Likelihood of receiving BRCA test	Which HR+ HER2- patient should be more likely to receive a BRCA test at diagnosis? Please assume that these	Number of attributes: 5<br>Number of levels per attribute: 2-3<br>Number of choice tasks: 9

Variable	Question	Format
	patients have no family history of breast cancer.	
**Section B1: Patient Characteristics and Treatment Selection – gBRCAm HR+ HER2- eBC**		
Treatment with Olaparib	Among all of your HR+ HER2- eBC patients who are gBRCAm and eligible for Olaparib, what percentage receive Olaparib?	Whole number
Factors influencing not receiving Olaparib	For your gBRCA HR+ HER2- eBC patients who are otherwise eligible for Olaparib and did not receive Olaparib, which of the following factors influenced the decision to **not** prescribe Olaparib?<br><br>(Multi-select; only asked of oncologists who reported fewer than 100% of eligible patients received olaparib)	Tumor stage suggests little benefit to patient<br>Patient unwillingness due to financial burden and/or lack of insurance coverage<br>Patient unwillingness due to treatment duration<br>Patient unwillingness due to other reason<br>Patient characteristics or situation changed such that the patient was no longer eligible for Olaparib<br>Comorbidity<br>A different treatment would have more benefit<br>A different treatment would have better tolerability<br>Need for genetic testing before prescribing<br>Other reason not associated with patient unwillingness
SCE: Patient characteristics influencing treatment selection in gBRCAm HR+ HER2- eBC	What are you most likely to prescribe as a treatment in the adjuvant setting with or without hormonal therapy for a gBRCAm HR+ HER2- patient with the following characteristics? Please assume the patient received neoadjuvant chemo with non-platinum chemo.	Number of treatment options: 5 or "no treatment or hormonal therapy only"<br>Number of attributes: 6<br>Number of levels per attribute: 2-3<br>Number of choice tasks: 12
**Section B2: Patient Characteristics and Treatment Selection – gBRCAm eTNBC**		
Treatment with Olaparib	Among all of your gBRCAm TNBC patients who are eligible for Olaparib, what percentage receive Olaparib?	Whole number
Factors influencing not receiving Olaparib	For your gRBCAm TNBC patients who are otherwise eligible for Olaparib and did not receive Olaparib, which of the following factors influenced the decision to **not** prescribe Olaparib?<br><br>(Multi-select; only asked of oncologists who reported fewer than 100% of eligible patients received olaparib)	Tumor stage suggests little benefit to patient<br>Patient unwillingness due to financial burden and/or lack of insurance coverage<br>Patient unwillingness due to treatment duration<br>Patient unwillingness due to other reason<br>Patient characteristics or situation changed such that the patient was no longer eligible for Olaparib<br>Comorbidity<br>A different treatment would have more benefit<br>A different treatment would have better tolerability<br>Need for genetic testing before prescribing<br>Other reason not associated with patient unwillingness
SCE: Patient characteristics influencing treatment selection in gBRCAm eTNBC	What are you most likely to prescribe as adjuvant therapy for a gBRCAm TNBC patient with the following characteristics? Please assume the patient received neoadjuvant IO plus chemotherapy.	Number of treatment options: 6 or "no treatment"<br>Number of attributes: 5<br>Number of levels per attribute: 2-3<br>Number of choice tasks: 12
**Section C: Treatment Characteristics and Treatment Selection**		
DCE: Influence of treatment	Which adjuvant treatment would you be more likely to prescribe to a BRCAm	Number of attributes: 7<br>Number of levels per attribute: 2

Confidential- Oracle Internal\Including External Recipients

Variable	Question	Format
characteristics on treatment selection in HR+ HER2- eBC	patient with HR+ HER2- eBC? Please assume that IDFS ranges from 86-89% and DRFS ranges from 88%-90% across both treatments.	Number of choice tasks: 9
DCE: Influence of treatment characteristics on treatment selection in eTNBC	Which treatment regimen would you be more likely to prescribe to a BRCAm patient with TNBC in early breast cancer?	Number of attributes: 6<br>Number of levels per attribute: 2-3<br>Number of choice tasks: 9
**Section D: Practice characteristics**		
Practice locale	Which of the following best describes the location of where you practice? *If you practice in multiple settings, please answer thinking about where you spend the majority of your time in direct patient care.*	A large city, population >500,000<br>A suburb near a large city, population between 100,000 and 500,000<br>Small city or town, population between 30,000 and 100,000<br>Rural area, population <30,000

BRCA, BReast CAncer gene; BRCAm, BRCA mutation; D.C., District of Columbia; DCE, discrete choice experiment; DRFS, distant relapse-free survival; eBC, early breast cancer; eTNBC, early triple-negative breast cancer; gBRCAm, germline BRCA mutation; HER2-, Human Epidermal Growth Factor Receptor 2-negative; HR+, hormone receptor-positive; IDFS, invasive disease-free survival; IO, immunotherapy; mBC, metastatic breast cancer; SCE, situational choice experiment; TNBC, triple-negative breast cancer.

Confidential- Oracle Internal\Including External Recipients

Supplementary Table 2. Physician characteristics (n=150)

		Oncologists
Age, years, mean (SD)		50.5 (8.6)
Gender, n (%)		
Male		113 (75.3)
Female		33 (22.0)
Other, such as non-binary, gender fluid, gender queer		3 (2.0)
Prefer not to answer		1 (0.7)
Race, n (%)		
Asian		35 (23.3)
Black or African American		6 (4.0)
White		97 (64.7)
Some other race or origin*		3 (2.0)
Prefer not to answer		9 (6.0)
Hispanic or Latino ethnicity, n (%)		
Yes		15 (10.0)
No		124 (82.7)
Prefer not to answer		11 (7.3)
US Census region, n (%)[†]		
Northeast		30 (20.1)
Midwest		35 (23.5)
South		48 (32.2)
West		36 (24.2)
Years in clinical practice (post-fellowship), mean (SD)		16.9 (6.8)
Primary practice setting, n (%)		
Academic		55 (36.7)
Community		95 (63.3)
Practice location, n (%)		
A large city, population > 500,000		74 (49.3)
A suburb population between 100,000 and 500,000, near a large city		58 (38.7)
Small city or town, population between 30,000 and 100,000		15 (10.0)
Rural area, population < 30,000		3 (2.0)
Percentage of time spent in direct patient care, mean (SD)		92.6 (7.8)
Number of eBC patients seen/treated in past 6 months	mean (SD)	104.4 (94.2)
	median (min-max)	67.5 (25-500)
Number of mBC patients seen/treated in past 6 months	mean (SD)	82.6 (75.1)
	median (min-max)	60 (15-400)
Number of TNBC patients seen/treated in past 6 months	mean (SD)	52.0 (65.6)
	median (min-max)	30 (4-400)
Number of patients with breast cancer referred for BRCA testing in past 6 months	mean (SD)	90.1 (89.1)
	median (min-max)	60 (4-500)

*No one identified as Native Hawaiian or other Pacific Islander or American Indian or Alaskan Native.
[†]Denominator (n = 149) excludes 1 oncologist who selected prefer not to answer when asked the state in which they primarily reside.

Confidential- Oracle Internal\Including External Recipients

eBC, early breast cancer; max, maximum; mBC, metastatic breast cancer; min, minimum; SD, standard deviation; TNBC, triple-negative breast cancer.

Confidential- Oracle Internal\Including External Recipients

Supplementary Table 3. Results for SCE-based questions: relative importance scores for patient characteristics in treatment selection of olaparib monotherapy for HER2- eBC by main practice setting

	**Relative importance scores in treatment selection of olaparib monotherapy**			
	**gBRCAm HR+/HER2- eBC***		**gBRCAm eTNBC†**	
**Attribute**	**Academic (n=55)**	**Community (n = 95)**	**Academic (n=55)**	**Community (n = 95)**
Age (45 years, 56 years)	21.6	9.7	4.6	4.0
Stage (IIB, III)	14.8	9.7	3.0	17.1
Ki-67 score (low, intermediate, high)	20.5	10.9	48.1	20.0
pCR status (pCR, no pCR)	8.0	4.5	25.1	39.5
Lymph node involvement‡	27.3	57.1	19.2	19.4
Oncotype Dx score (26, 50)	8.0	8.1	n/a	n/a

Note: Relative importance scores sum to 100. Relative importance scores are interpreted as the percentage of total importance (difference between most influential versus least influential level of the attribute) within a treatment option (i.e., olaparib monotherapy in this table). Higher scores indicate that the change within a patient characteristic had greater influence on choosing olaparib monotherapy. Relative importance scores are ratio-scaled; hence, an attribute with a relative importance of 20 is twice as important as an attribute with a relative importance of 10. Attribute levels are presented in parentheses.

*Physicians were prompted with: *What are you most likely to prescribe as a treatment in the adjuvant setting with or without hormonal therapy for a gBRCAm HR+ HER2- patient with the following characteristics? Please assume the patient received neoadjuvant chemo with non-platinum chemo.*

†Physicians were prompted with *What are you most likely to prescribe as adjuvant therapy for a gBRCAm TNBC patient with the following characteristics? Please assume the patient received neoadjuvant IO plus chemotherapy.*

‡Attribute levels for lymph node involvement varied for the two settings. In the HR+/HER2- eBC setting, attribute levels were: no lymph node involvement, 1 to 3 positive lymph nodes, 4 or more positive lymph nodes. In the eTNBC setting, attribute levels were pN0 < pT2, pN0 ≥ pT2, ≥ pN1.

CDK4/6i, Cyclin-Dependent Kinases 4 and 6 inhibitor; eBC, early breast cancer; eTNBC, early triple-negative breast cancer; gBRCAm, germline BRCA mutation; HER2-, Human Epidermal Growth Factor Receptor 2-negative; HR+, hormone receptor-positive; IO, immunotherapy; pCR, pathological complete response; SCE, situational choice experiment.

Confidential- Oracle Internal\Including External Recipients

2 Supplementary Figures

Supplementary Figure 1. Example choice task from the Discrete Choice Experiment (DCE) evaluating the influence of patient characteristics on the likelihood of a patient with HR+/HER2- eBC with no family history of breast cancer receiving BRCA testing at diagnosis

Which HR+ HER2- patient should be more likely to receive a BRCA test at diagnosis?
Please assume that these patients have **no family history** of breast cancer.

**Patient 1**	**Patient 2**
Age <35	Age ≥65
Pre-menopausal	Post-menopausal
No lymph node involvement	4 or more positive lymph nodes
Tumor size <1 cm	Tumor size >5 cm
Breast conversation is prioritized	Breast conversation is not prioritized

DCE, discrete choice experiment; eBC, early breast cancer; HER2-, Human Epidermal Growth Factor Receptor 2-negative; HR+, hormone receptor-positive.

Confidential- Oracle Internal\Including External Recipients

Supplementary Figure 2. Example choice tasks from the DCEs evaluating the influence of treatment characteristics on the selection of treatment for a BRCAm patient with HR+/HER2- eBC **(A)** and eTNBC **(B)**.

A

Which adjuvant treatment would you be more likely to prescribe to a BRCAm patient with HR+ HER2- eBC?
Please assume that IDFS ranges from 86-89% and DRFS ranges from 88%-90% across both treatments.

Treatment 1	Treatment 2
92% OS at 3 years	Unknown OS at 3 years
Oral treatment BID for 1 year	Oral treatment BID for 2 years
Discontinuation rate: 10%	Discontinuation rate: 19%
5% risk of G3/4 neutropenia	19% risk of G3/4 neutropenia
8% risk of G3/4 diarrhea	<1% risk of G3/4 diarrhea
2% risk of G3/4 anemia	9% risk of G3/4 anemia
28% risk of all grade nausea	57% risk of all grade nausea

B

Which treatment regimen would you be more likely to prescribe to a BRCAm patient with TNBC in early breast cancer?

Regimen 1	Regimen 2
79% OS at 5 years<br>70% IDFS at 5 years	90% OS at 3 years<br>87% DRFS at 3 years
Oral treatment	IV treatment
24% risk of G3/4 AEs	77% risk of G3/4 AEs
17.6% risk of all grade diarrhea	30.4% risk of all grade diarrhea
<1% risk of G 3/4 immune system related side effects that can affect the stomach, intestines, lungs, liver or other organs and require IV steroids and hospitalization	12% risk of G 3/4 immune system related side effects that can affect the stomach, intestines, lungs, liver or other organs and require IV steroids and hospitalization
<1% risk of G 3/4 hand foot syndrome	11% risk of G 3/4 hand foot syndrome

AEs, adverse events; BRCAm, BRCA mutation; DCE, discrete choice experiment; DRFS, distant relapse-free survival; eBC, early breast cancer; eTNBC, early triple-negative breast cancer; G3/4; grade 3/4; HER2-, Human Epidermal Growth Factor Receptor 2-negative; HR+, hormone receptor-positive; IDFS, invasive disease-free survival; IV, intravenous; OS, overall survival.

Confidential- Oracle Internal\Including External Recipients

Supplementary Figure 3. Example choice tasks from the SCE-based questions evaluating the influence of patient characteristics on the selection of adjuvant treatment for a gBRCAm patient with HR+/HER2- eBC **(A)** and eTNBC **(B)**.

A

What are you most likely to prescribe as a treatment in the adjuvant setting with or without hormonal therapy for a gBRCAm HR+ HER2- patient with the following characteristics? *Please assume the patient received neoadjuvant chemo with non-platinum chemo.*

Patient A	Patient B	Patient C
**Age 56**<br>**Stage IIB**<br>**Ki-67 low**<br>**pCR**<br>**No lymph node involvement**<br>**OncotypeDx score 26**	**Age 56**<br>**Stage IIB**<br>**Ki-67 intermediate**<br>**No pCR**<br>**1 to 3 positive lymph nodes**<br>**OncotypeDx score 26**	**Age 45**<br>**Stage III**<br>**Ki-67 high**<br>**No pCR**<br>**4 or more positive lymph nodes**<br>**OncotypeDx score 50**
Olaparib only	Olaparib only	Olaparib only
CDK4/6i only	CDK4/6i only	CDK4/6i only
Olaparib + CDK4/6i	Olaparib + CDK4/6i	Olaparib + CDK4/6i
Olaparib followed by CDK4/6i	Olaparib followed by CDK4/6i	Olaparib followed by CDK4/6i
CDK4/6i followed by Olaparib	CDK4/6i followed by Olaparib	CDK4/6i followed by Olaparib
No treatment	No treatment	No treatment

B

What are you most likely to prescribe as adjuvant therapy for a gBRCAm TNBC patient with the following characteristics? *Please assume the patient received neoadjuvant IO plus chemotherapy.*

Patient A	Patient B	Patient C
**Age 56**<br>**Stage IIB**<br>**Ki-67 low**<br>**≥ pN1**<br>**pCR**	**Age 56**<br>**Stage IIB**<br>**Ki-67 intermediate**<br>**pN0 with ≥ pT2**<br>**No pCR**	**Age 45**<br>**Stage III**<br>**Ki-67 high**<br>**pN0 with ≥ pT2**<br>**No pCR**
Olaparib only	Olaparib only	Olaparib only
Olaparib + IO	Olaparib + IO	Olaparib + IO
IO only	IO only	IO only
Olaparib followed by IO	Olaparib followed by IO	Olaparib followed by IO
IO followed by Olaparib	IO followed by Olaparib	IO followed by Olaparib
Capecitabine only	Capecitabine only	Capecitabine only
No treatment	No treatment	No treatment

CDK4/6i, Cyclin-Dependent Kinases 4 and 6 inhibitor; eTNBC, early triple-negative breast cancer; gBRCAm, germline BRCA mutation; HER2-, Human Epidermal Growth Factor Receptor 2-negative; HR+, hormone receptor-positive; IO, immunotherapy; pCR, pathological complete response; SCE, situational choice experiment; TNBC, triple-negative breast cancer.

Confidential- Oracle Internal\Including External Recipients
